# Alpha-2 agonism in the locus coeruleus impairs learning driven by negative prediction error

**DOI:** 10.1038/s41386-025-02092-5

**Published:** 2025-04-13

**Authors:** Ashleigh K. Brink, Simon K. C. Lui, Laura H. Corbit

**Affiliations:** 1 Department of Cell and Systems Biology, University of Toronto, Toronto, ON Canada; 2 Department of Psychology, University of Toronto, Toronto, ON Canada

**Keywords:** Psychology, Extinction

## Abstract

Refining previous learning when environmental contingencies change is a critical adaptive function. Studies have shown that systemic noradrenaline (NA) manipulations, as well as optogenetic manipulations of the locus coeruleus (LC), the primary source of forebrain NA, can improve long-term retention of appetitive extinction. To determine whether the contribution of NA is specific to extinction or extends to other forms of learning where reward is less than expected, we suppressed LC activity with clonidine, an α_2A_-adrenergic receptor agonist, in two tasks: compound extinction, where two previously rewarded cues are presented together and no longer rewarded, and overexpectation, where animals are presented with two previously rewarded cues but receive a single reward rather than the expected two. In compound extinction, we found no differences between groups in training, extinction, or a spontaneous recovery test. However, animals that received clonidine reacquired responding to the previously extinguished cue significantly faster than saline-treated animals, suggesting weakened extinction learning. In overexpectation testing, the saline group responded significantly less to a stimulus that had undergone overexpectation relative to a control stimulus, indicating that they had recalibrated their estimation of reward magnitude following training where reward was less than expected. In contrast, clonidine-treated animals did not differ in responding to the overexpectation versus control stimuli, suggesting that clonidine impaired learning resulting from overexpectation. These results demonstrate that activity of the LC is important for learning to reduce responding in both extinction and overexpectation paradigms.

## Introduction

Detecting and encoding relationships between events as well as modifying previous learning when confronted with new information is essential for adaptive control of behaviour. For example, when a previously reinforced stimulus or response is no longer reinforced, the previously learned behaviour should be reduced or extinguished, allowing resources to be allocated elsewhere. However, there is substantial evidence that the expression of extinction is often not permanent, and the original learning can return [1–3]. When the originally learned behaviours are unwanted, or no longer appropriate, this presents a challenge for behaviour modification, particularly in the clinical context of extinction-based therapies. Thus, there is interest in strategies to enhance the retention of extinction.

Contemporary theories suggest that learning is driven by the discrepancy between what is expected based on available predictors, and what actually occurs [4–6]. This discrepancy is referred to as prediction error and the larger the error, the more learning that should result on a given trial. Two paradigms exploit this idea: compound extinction and overexpectation. In either task, multiple stimuli are trained independently, and each comes to predict the reinforcer it has been paired with. When such stimuli are then presented simultaneously, each predicts the outcome and so together, double the outcomes are predicted. When this expectation is not met, either in extinction when no reinforcer is delivered, or in overexpectation, when a smaller than expected reinforcer is delivered, a negative prediction error is produced leading to a decrease in the associative strength, or predictive power, of each stimulus across trials [5, 7–10].

Extinction and overexpectation provide methods for reducing previously learned behaviours, however, both are subject to spontaneous recovery and renewal [3, 11–13], suggesting that their expression can be disrupted by the passage of time or changes in the test environment, and that they rely on new inhibitory learning that competes for behavioural control, rather than the erasure of the original learning. Interestingly, compound extinction has been shown to be more resistant to spontaneous recovery than extinction of a single stimulus [14–17], and augmenting noradrenergic activity has been found to enhance appetitive extinction [15–17]. Thus, these strategies have potential for enhancing behavioural change. Nonetheless, the neural and pharmacological bases of these effects are not well understood.

Endogenously, NA is released when an expected reward is omitted [18, 19]. The likely source of this NA is the locus coeruleus (LC) which sends noradrenergic projections throughout the forebrain [20–22], facilitating plasticity in diverse projection regions [21, 23–28]. Electrophysiological and fibre photometry recording studies have observed that LC neurons resume firing in early extinction trials [29–31] However, evidence is mixed [32–35]. Recent studies using chemogenetic or optogenetic LC stimulation are similarly conflicting [26, 36–38], perhaps due to differences in the effects of tonic vs. phasic firing [22, 39], previously unrecognised functional heterogeneity amongst LC projections [21, 26], or differences in the intensity or duration of LC stimulation and interactions with arousal state and corresponding levels of endogenous NA. Ultimately, different levels of LC activation may produce different effects on extinction learning.

The current study sought to better understand the role of the LC in extinction learning. To do so, we infused clonidine, an alpha-2-receptor agonist into the LC to inhibit LC-NA neurons. We hypothesised that clonidine acting on local autoreceptors during extinction of food seeking would attenuate extinction learning. Furthermore, as emerging evidence now suggests that the LC plays a broader role in prediction error signalling [23, 24, 31, 40, 41] we also examined whether the LC contributes to another example of learning driven by a negative prediction error; overexpectation, where reinforcement is still delivered, but is smaller than anticipated based on the stimuli presented. We infused clonidine prior to overexpectation trials, hypothesising that attenuating LC-NA activity would impair the animals’ ability to update their previous learning in this task.

## Materials and methods

### Subjects

Sixty-three (59 male, 4 female) 8-week-old Long-Evans rats served as subjects (Charles River, St. Constant, QC, Canada). Housing was identical to our previous studies [38]. Experimental procedures were performed in accordance with guidelines from the Canadian Council on Animal Care and approved by the University of Toronto Animal Care Committee.

### Apparatus

Rats were trained and tested in 8 identical behavioural chambers (Med Associates, Fairfax, VT, USA) described in [38]. Each chamber contained two key lights situated above retractable levers and a magazine where 45-mg grain pellets could be delivered (F0165; BioServ, Flemington, NJ, USA). A white noise, 5-Hz clicker, and a 2900 Hz tone were used as auditory stimuli in addition to the visual key lights. The auditory stimuli were adjusted to 80 dB in the presence of background noise of 60 dB provided by a ventilation fan. Chambers were illuminated by a 3-W, 24-V house light. All experimental events were controlled and recorded with MED-PC V software (Med Associates).

### Stereotaxic surgery

Surgical procedures have been described elsewhere [38]. Briefly, stereotaxic surgery was performed to implant guide cannulae (26 G; HRS Scientific, Anjou, QC, Canada) to bilaterally target the LC (AP: −10.0 mm, ML: ±1.3 mm, DV −7.4 mm, relative to bregma and skull). Guide cannula were positioned 3.0 mm above the LC and secured in place with dental cement. The internal cannula used for drug delivery extended 3.0 mm past the guide cannula to reach the final DV coordinates.

### Behavioural training and testing

All rats were trained in a discriminated operant task where lever-pressing in the presence of a stimulus resulted in food reward. Clonidine was delivered prior to key extinction or overexpectation sessions as outlined below.

### Magazine and lever training

Following surgical recovery, rats were food restricted and maintained at 90% of their free feeding weight. Rats received one 30-min magazine training session where pellets were delivered according to a random time 60-s schedule (RT-60s). Next, rats were trained to lever-press for food reward. Each lever press delivered a pellet until 50 pellets were earned.

### Discrimination training

Next, rats were trained in a discriminated operant procedure in which responding during stimulus presentations resulted in pellet delivery, whereas responding in the absence of a stimulus had no programmed consequences. Each session contained eight, 20-s presentations of a light (both key lights illuminated), white noise, or clicker stimulus (24 trials total). Responding was reinforced according to random ratio (RR) schedules that increased across days (3 days of each RR1, RR2, and RR4). Trial order was pseudo-randomly determined and the intertrial interval (ITI) averaged 90 s. Rats received two mock infusions prior to the first day of drug delivery.

### Drug infusions

On infusion days, animals received a bilateral infusion of saline or clonidine (0.6 μg dissolved in 0.2 μL saline per hemisphere; Sigma-Aldrich, Oakville, ON, Canada) into the LC via internal cannulae (33 G; HRS Scientific). This dose was selected based on pilot experiments (see Supplemental Materials, Figure S1). A total volume of 0.2 μL was infused over 2 min, and the cannulae remained in place for an additional 1 min to allow diffusion away from the tip. The delay between drug infusion and testing ranged from 5 to 20 min.

#### Experiment 1: compound extinction

Based on our previous pharmacological studies implicating noradrenaline in the ability of compound extinction to enhance subsequent expression of extinction learning [15], we examined whether LC inhibition would impair this effect. The experimental design is summarised in Fig. 2A and follows that of several experiments that demonstrate that after extinguishing stimuli individually, presenting them in compound produces greater responding, potentially because the compound reintroduces a prediction error, which in turn leads to further learning and less responding at test [14, 15, 42]. Following discrimination training, rats (n = 24) were given 2 sessions of extinction that were identical to the training sessions, except that lever presses were not reinforced. Prior to a third extinction session, rats were assigned to saline (n = 12) or clonidine (n = 12) groups matched for response rates on the final day of training. Following infusions, both groups received six compound stimulus presentations consisting of the light stimulus coincident with either the white noise or the clicker stimulus (counterbalanced). Testing was conducted four weeks later and consisted of six presentations of the auditory stimulus that was presented in the final extinction session as part of the compound, but delivered alone at test (i.e., white noise or clicker). Based on the previous observation that an extinguished response can recover rapidly when the response is reinforced again [43], providing additional evidence that extinction does not erase the original learning, the following day, rats were tested for reacquisition of responding where lever-pressing during stimulus presentations was again reinforced according to a RR4 schedule.

#### Experiment 2: overexpectation

Next, we tested the effects of LC inactivation on overexpectation in a separate cohort of rats (n = 21). In addition to the initial discrimination training described above, rats underwent 3 days of training on a RR5 schedule followed by 3 on a RR10 schedule. The following day overexpectation trials were introduced. These sessions contained 12 trials of a single auditory stimulus (clicker or white noise; counterbalanced) intermixed with 12 trials of a compound stimulus (e.g., light + the other auditory stimulus; white noise or clicker; see Fig. 3A). For each trial, rats could earn a maximum of one pellet if the lever was pressed during stimulus presentation. Rats were assigned to saline (n = 10) or clonidine (n = 11) groups based on response rates in training and received a total of four overexpectation sessions. Infusions of either saline or clonidine (0.6 μg) were given prior to each of these sessions. On the following day, rats were given a nonreinforced test containing four trials of each auditory stimulus (eight trials total) to determine whether previous clonidine treatment impacted the learning that occurred during overexpectation. No infusions were given during testing.

#### Experiment 3: compound extinction and comparison to a control compound

This experiment was designed to address potential alternative explanations and differences between Experiments 1 and 2. First, in overexpectation a single stimulus continued to be reinforced throughout drug treatment, however, responding to this stimulus was lower in the clonidine group. Although in Experiment 1 there were no group differences in responding during the spontaneous recovery test, and the clonidine group responded more during reacquisition, there was no comparison of the extinguished stimulus to one that was always reinforced leaving some uncertainty as to whether the effects of clonidine were specific to extinction. To address this an additional stimulus was added that continued to be reinforced during sessions where other stimuli underwent extinction (Z; see Fig. 4A). Second, while elsewhere compound extinction has been shown to deepen extinction compared to equivalent extinction of a single stimulus, it is not clear whether this effect is due to additional prediction error introduced by the compound or simply the novelty of the compound itself. The following experiment trained an additional stimulus that was never reinforced. On the drug day, there were two types of compounds: AX, comprised of two previously reinforced stimuli as in Experiment 1, and BY, comprised of a previously reinforced stimulus and the stimulus that was never reinforced. The BY compound controls for the novel introduction of a stimulus compound without augmenting the prediction error. Finally, we tested retention of extinction 24 h after drug treatment to match the test conditions for overexpectation. Eighteen rats underwent discrimination training that included two additional stimuli (flashing keylights and a tone). One of the light stimuli (solid or flash; counterbalanced) was never reinforced whereas the tone was always reinforced. Each session had 6 trials of each stimulus (30 trials total). Next, there were two extinction sessions where all stimuli except the tone were extinguished. Prior to the third extinction session rats received an infusion of saline (n = 9) or clonidine (0.6 μl, n = 9) and received 3 trial types (6 trials each); AX where either the noise or clicker were paired with the previously reinforced light; BY where the alternate auditory stimulus was paired with the never reinforced light; and tone (Z) trials which were reinforced as in training. The following day, rats were given a retention test comprised of 3 nonreinforced trials of each of the auditory stimuli.

#### Histology

After testing, rats were overdosed with isoflurane and transcardially perfused with 0.1 M phosphate-buffered saline (PBS) followed by 10% phosphate-buffered formalin. Cryoprotected brains were frozen and sliced using a cryostat (40-μm; CM1860; Leica, Buffalo Grove, IL, USA), mounted, and stained with cresyl violet to verify cannulae placements.

## Results

### Histology

**Fig. 1:**
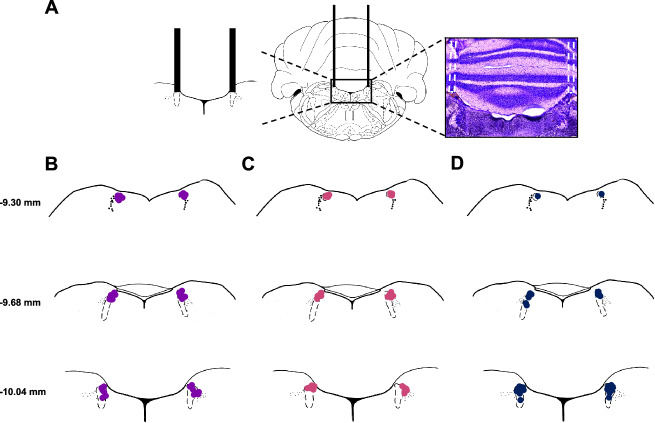
Histology. **A** Schematic of the guide cannula placement (AP: −10.0 mm; ML: ±1.3 mm; DV −7.4 mm) directly above the LC (left) and a representative image of cannula placement after cresyl violet staining (right). Diagram of internal cannula tip placement within the LC for all animals included in the compound extinction experiment (**B**); overexpectation experiment (**C**); and experiment 3 (**D**). Coordinates and images are adapted from Paxinos and Watson 5th Edition [57].

Nine rats (8 males, and 1 female) were excluded from analyses due to off-target cannula placement. Placements for rats included in the behavioural analyses are displayed in Fig. 1. Final group sizes were as follows; compound extinction saline (n = 12) and clonidine (n = 9); overexpectation saline (n = 9) and clonidine (n = 9), Experiment 3; saline (n = 7) and clonidine (n = 8).

### Compound extinction

**Fig. 2:**
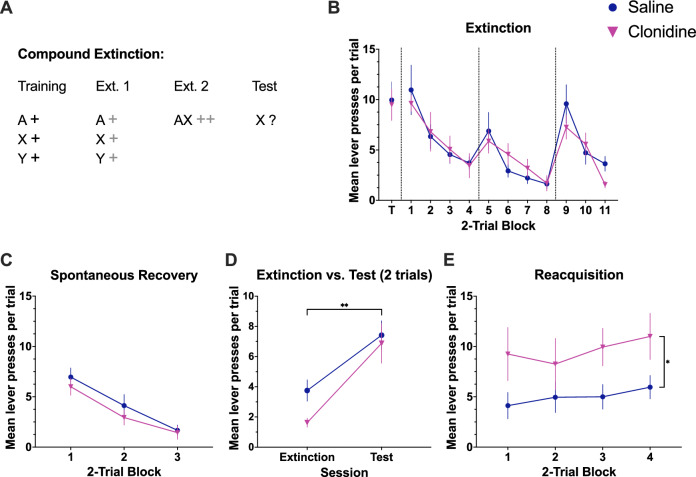
Inhibition of the LC with clonidine attenuates compound extinction allowing rapid reacquisition of responding. **A** Summary of the training and testing procedures used in compound extinction. A represents the light stimulus (two key lights illuminated on the chamber wall). X and Y represent the two auditory stimuli, clicker and white noise, counterbalanced. A black ‘+’ represents delivery of a single pellet whereas grey a ‘+’ represents omission of an expected pellet. Following training, rats received two extinction sessions where all stimuli were presented but no pellets were delivered. In the third extinction session stimuli A and X were presented simultaneously as a compound (6 trials) and no pellets were delivered. Saline or clonidine was infused prior to this final extinction session (rightmost portion of panel **B**). Responding to stimulus X was tested drug-free four weeks later. **B** The vertical dotted lines indicate breaks between days. Groups showed equivalent responding on the final day of training (T; p = 0.855). Responding in both groups decreased across the first two days of extinction [blocks 1-8; p = 0.001]. Further, we observed an increase in responding from Day 2 of extinction, when stimuli were presented separately, to Day 3 when two stimuli were presented together as a compound [blocks 9–11; p = 0.007], indicating the presence of summation. There were no significant differences between groups or day x group interactions [p’s > 0.05]. There was no significant difference between groups in the test conducted 4 weeks after the compound extinction training session (**C**; p = 0.478). **D** To confirm spontaneous recovery, we compared the final two trials of extinction to the first two trials of testing and observed an increase in responding across time [p = 0.001] but no difference between groups [p = 0.210]. **E** In a test of reacquisition, we found that animals in the saline group were slower to resume responding compared to the clonidine group that rapidly resumed responding when reward was again available [p = 0.019], consistent with weakened retention of extinction learning in the clonidine group. Dots represent group means for 2-trial blocks. Error bars represent the standard error of the mean. Clonidine is represented by triangular data points. Saline is represented by circular data points. *, **, *** = p < 0.05, p < 0.01, p < 0.001.

Clonidine and saline groups showed similar performance on the final day of training [Fig. 2B; F(1, 18) = 0.034, p = 0.855]. Responding in both groups decreased across the first two days of extinction evidenced by an effect of day [Fig. 2B; F(1, 18) = 37.395, p = 0.001], but no effect of group [F(1, 18) = 0.015, p = 0.903] and no interaction between these factors [F(1, 18) = 0.398, p = 0.536]. Evidence of summation when the compound was introduced was found in the increase in responding from Day 2 of extinction, when stimuli were presented separately, to Day 3 when two stimuli were presented together [effect of day; F(1, 18) = 9.084, p = 0.007]. This increase was similar for the two groups as there was no effect of group or day x group interaction [Fs < 1]. To examine spontaneous recovery, we compared the final two trials of extinction to the first two trials of testing which demonstrated an increase in responding across time [Fig. 2D; (1, 18) = 32.763, p = 0.001], but no effect of group [F(1, 18) = 1.687, =0.210] and no time x group interaction [F(1, 18) = 1.033, p = 0.323]. Because responding during testing was low which may have masked group differences, to further assess retention of extinction, we compared reacquisition of responding in a session where lever-pressing again earned reward. Two animals failed to earn any reward in this session (one from each group) and were excluded from further analyses. The previously saline-treated animals were slower to resume responding consistent with stronger extinction compared to the previously clonidine-treated group that rapidly resumed responding when reward was again available [Fig. 2E (1, 16) = 6.808, p = 0.019].

### Overexpectation

**Fig. 3:**
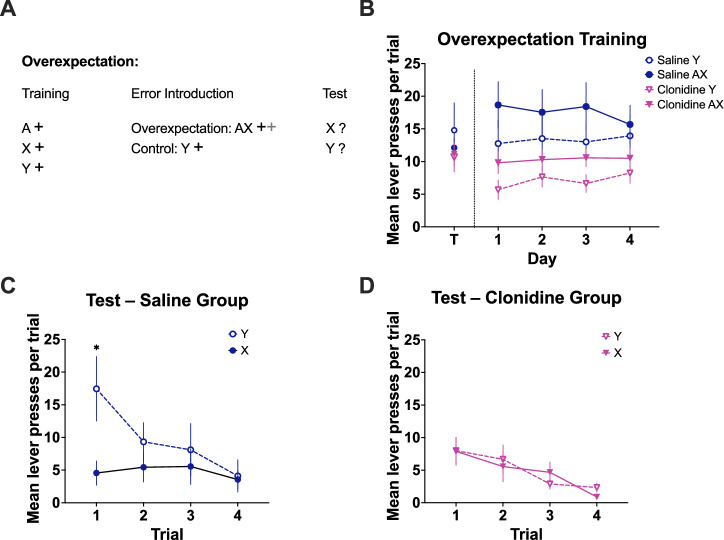
Inhibition of the LC with clonidine attenuates learning driven by a reduction in reward in the overexpectation task. **A** Summary of the training and testing procedures used in overexpectation. A represents the light stimulus (two key lights illuminated on the chamber wall). X and Y represent the two auditory stimuli, clicker and white noise, counterbalanced. A black ‘+’ represents delivery of a single pellet whereas a grey ‘+’ represents omission of expected pellets. Saline or clonidine was infused prior to the four sessions where overexpectation trials were introduced. Responding to stimulus X versus Y was tested drug-free the following day. **B** The vertical dotted lines indicate breaks between days. On the final day of training, there was no difference between the stimulus that would be presented alone (Y) and the stimulus that would be part of the overexpectation compound stimulus (X) [p = 0.542], nor the saline and clonidine groups [p = 0.448]. There was no interaction between these factors [p = 0.397]. On the first day of overexpectation training, responding was significantly elevated in compound-stimulus (overexpectation) trials relative to single-stimulus trials [p = 0.001]. By the fourth day of overexpectation training, responding to the compound and single stimuli had converged, with no effect of stimulus [p = 0.072], nor group [p = 0.118], and no interaction between these factors [p = 0.809]. **C** Early in the test, the stimulus that was previously part of the compound (X) elicited less responding than the stimulus that was always presented alone (Y) for the saline group [stimulus x trial interaction; p = 0.037]. Simple effects analyses further confirmed a significant difference between stimuli at trial 1 [p = 0.032] but not at trial 4 [p = 0.757]. **D** This difference was absent in the clonidine group, with no significant difference in responding between stimuli (p = 0.325). *, **, *** = p < 0.05, p < 0.01, p < 0.001.

On the final day of training were no effects of stimulus [Fig. 3B; to-be single (Y), to-be overexpectation (X); F(1, 16) = 0.338, p = 0.542], or group (clonidine, saline; F(1, 16) = 0.605, p = 0.448), and no interaction between these factors [F(1, 16) = 0.757, p = 0.397]. On the first day of overexpectation, the compound stimulus trials produced robust summation. Greater responding was evident in compound (overexpectation) trials relative to single stimulus trials [Fig. 3B; F(1, 16) = 46.520, p = 0.001]. The clonidine group responded somewhat less overall resulting in a marginal effect of group [F(1, 16) = 4.327, p = 0.054] but there was no interaction between stimulus and group indicating elevated responding to the compound stimulus in both groups [F(1, 16) = 1.477, p = 0.242].

Over the course of overexpectation training, responding to the compound and single stimuli converged. By the fourth day of overexpectation, there was no longer an effect of stimulus [Fig. 3B; F(1, 16) = 3.717, p = 0.072], nor group [F(1, 16) = 2.731, p = 0.118], and no interaction between these factors [F(1, 16) = 0.061, p = 0.809].

The data from the test session demonstrate that for the saline group, the stimulus that was previously part of the compound evoked less responding than the stimulus that was always presented alone but that this difference was absent for animals that previously received clonidine [Fig. 3C, D]. ANOVA demonstrated no main effect of stimulus [F(1, 16) = 2.369, p = 0.143] or stimulus x group interaction [F(1, 16) = 1.981, 0.143]. There was however an effect of trial [F(1, 16) = 10.522, p = 0.001], a stimulus x trial interaction [F(3, 16) = 2.834, p = 0.048] and a stimulus x trial x group interaction [F(3, 48) = 3.135, p = 0.034]. To explore the 3-way interaction we examined the effects of stimulus and trial separately for each group. For the saline group, there was no effect of stimulus [F(1, 8) = 2.281, p = 0.169], but an effect of trial [F(3, 24) = 7.648, p = 0.001] and a stimulus x trial interaction [F(3, 24) = 3.326, p = 0.037]. As suggested by Fig. 3C, early in the test the stimulus that was previously part of the compound evoked less responding than the stimulus that was always presented alone. Simple effects analyses confirmed an effect of stimulus on trial 1 [F(1, 8) = 6.762, p = 0.032] but responding to the two stimuli was equivalent by trial 4 [F(1, 8) = 0.103, p = 0.757]. For the clonidine group (Fig. 3D), there was an effect of trial, with responding decreasing across trials [F(3, 24) = 4.023, p = 0.019], but there was no effect of stimulus [F(1, 8) = 0.090, p = 0.772] and no stimulus x trial interaction [F(3, 24) = 1.216, p = 0.325] suggesting responding to the two stimuli was equivalent throughout the test session. Although responding was low in the clonidine group, on the first test trial responding to the previous compound was higher in this group compared to the saline group suggesting the drug effect is not simply a floor effect [F(1, 16) = 6.101, p = 0.025].

### Compound extinction 2

**Fig. 4:**
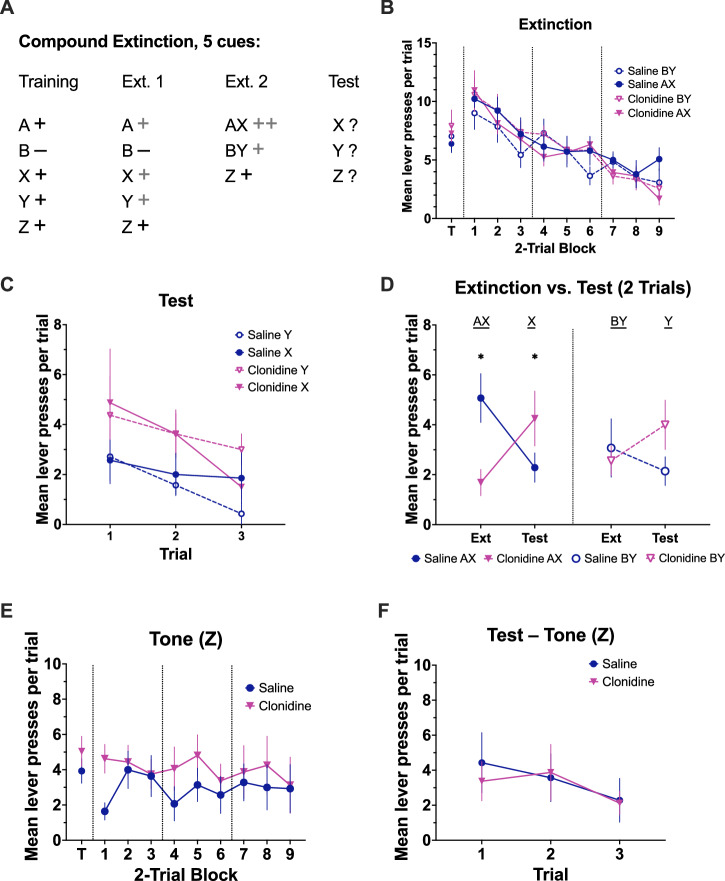
The compound extinction effect is not explained by the novelty of a stimulus compound. **A** Summary of the training and testing procedures used in Experiment 3. A and B represent the light stimuli (two key lights either flashing or continuously illuminated; counterbalanced). X and Y represent the two auditory stimuli, clicker, and white noise, counterbalanced. Z represents a tone stimulus that was always reinforced up until testing. A black ‘+’ represents delivery of a single pellet whereas a grey ‘+’’ represents omission of predicted pellets. ‘-’ indicates a stimulus that was never reinforced and thus does not predict an outcome. Following training, rats received two sessions of extinction where stimuli A, B, X, and Y were not reinforced. Stimulus Z continued to be reinforced until the test session. Clonidine or saline was infused prior to the third extinction session which contained two types of compound stimulus trials: AX and BY (nonreinforced), as well as Z trials (reinforced). Responding to X, Y, and Z was tested the following day. **B** The vertical dotted lines indicate breaks between days. There were no group or stimulus differences at the end of training. Responding to X and Y decreased in both groups across the first two days of extinction [p < 0.001]. **C** Test data across trials. **D** To assess whether the AX was more effective in promoting extinction than the control BY and whether this was altered by clonidine we compared the final two trials of extinction to the first two trials of the test. While there were no main effects [Fs < 1], there were significant day x group [F(1,13) = 6.113, p = 0.028] and stimulus x day x group [F(1,13) = 3.831, p = 0.035] interactions. In the final trials of extinction the saline group responded more to the excitatory compound AX than the clonidine group [p = 0.0018] whereas responding to the BY was similar [p = 0.3458]. In contrast, at test the clonidine group responded more to stimulus X than the saline group [p = 0.048] whereas there was no group difference in responding to Y [p = 0.4245]. **E**, **F** No group differences were observed in responding to the tone at any phase.

Clonidine and saline groups showed similar performance on the final day of training [Fig. 4B; F(1, 14) = 0.182, p = 0.677]. Responding to X and Y stimuli did not differ [no effect of stimulus; F(1, 14) = 1.315, p = 0.271; no stimulus x group interaction F < 1]. Responding to X and Y decreased in both groups across the first two days of extinction [Fig. 4B; day: F(1, 14) = 17.225, p = 0.001; no effects of group, stimulus, and no interactions; all Fs < 1]. To assess whether AX was more effective in promoting extinction than BY and whether this was altered by clonidine we compared the final two trials of extinction to the first two trials of the test. While there were no main effects [Fs < 1], there were significant day x group [F(1, 13) = 6.113, p = 0.028] and stimulus x day x group [F(1, 13) = 3.831, p = 0.035] interactions. Pairwise comparisons found that during extinction the saline group responded more to AX than the clonidine group [Fig. 4D; t(13) = 3.486. p = 0.0018] whereas responding to BY was similar [t(13) = 0.405, p = 0.3458]. In contrast, at test the clonidine group responded more to stimulus X than the saline group [t(13) = 1.794, p = 0.048] whereas there was no group difference in responding to Y [t(13) = 0.194, p = 0.4245]. No group differences were observed in responding to the tone (Z; Fig. 4E, F) at any phase [largest F = 1.639, p = 0.223] suggesting that clonidine did not affect responding to a reinforced stimulus.

## Discussion

These results extend previous findings using systemic pharmacological treatments [15] and optogenetic manipulation of the LC [26, 38] and provide further evidence that activity of the LC contributes to the retention of appetitive extinction. Importantly, these data demonstrate that LC activity, and presumed NA release, also contribute to learning that occurs in the overexpectation task, where reward continues to be delivered, but is less than expected based on the predictive stimuli that are present [5, 7]. These findings are important because they suggest NA is not uniquely involved in learning about the absence of a reinforcer, but its effects extend to other types of learning driven by a negative prediction error.

From a behavioural perspective, evidence of summation, measured as greater responding to a stimulus compound than to the elements of that compound, supports the idea that animals base their expectations on the combination of predictions from the stimuli available. Importantly, we observed significant summation in the early trials of each task after which responding gradually decreased as predicted by the Rescorla-Wagner model [5] and consistent with the idea that the animals’ prediction for the compound is gradually updated as they gain experience with the new reinforcer (none in extinction, or smaller in overexpectation). Inclusion of a control compound in Experiment 3 further suggest these effects are related to prediction rather than the novelty of the compound trials. Furthermore, the finding that clonidine did not affect responding to a reinforced stimulus makes it unlikely that effects on extinction or overexpectation were due to non-specific effects of the drug. Although there was no group difference in responding to the stimulus extinguished with a non-predictive stimulus (Y) there was some evidence of recovery of responding to this stimulus in the clonidine group. This is not necessarily surprising as that stimulus did undergo extinction which requires new learning [2] that can be augmented by LC activity [38] and so blocking LC activity may be expected to reduce extinction retention, albeit to a lesser extent in this condition. Future experiments with more extinction trials for each stimulus (as in Experiment 1) may reveal greater differences in responding to the AX and BY compounds across extinction and more pronounced effects at test. Further, while testing was performed 24 h after the last extinction session to allow comparison to Experiment 2, overall responding was low. Group differences may have been more apparent had we allowed a longer delay before testing to permit spontaneous recovery as in Experiment 1 and other previous studies [14, 15].

The presumed reduction in NA release following clonidine likely affected plasticity at LC targets [26, 44] which could account for the effects seen at test. As the LC projects broadly throughout the brain, future studies should examine which of these targets are responsible for the effects of LC inactivation although the amygdala and infralimbic cortex are strong candidates [1, 26, 44–46]. These results may appear at odds with our recent demonstration that optogenetic inhibition of the LC did not impair extinction [38]. However, in that study, the halorhodopsin virus was expressed in only ~55% of neurons and the remaining population may have been sufficient to support extinction learning. The clonidine treatment here may have had a more complete effect.

While multiple studies have now implicated NA in extinction [15–19, 26, 38, 44], here we explored whether NA is also important for overexpectation. Critically, during testing, saline-treated animals responded less to the overexpectation stimulus consistent with the predicted loss of associative strength across compound trials compared to the single stimulus. This difference was not seen in animals previously treated with clonidine suggesting that inhibition of the LC interfered with the learning that occurred for saline-treated animals during overexpectation training. These results suggest that NA also contributes to learning about a reduced reward, but where reinforcement is still present, thus extending its role beyond extinction. The overexpectation results are also important because this procedure provides an opportunity to reduce the influence of predictive stimuli under circumstances where omitting the outcome entirely may not be possible or practical (e.g., in cases of obesity or eating disorders where the individual must continue to eat). Discovery that LC activity contributes to this effect suggests that enhancing noradrenaline could enhance learning in this paradigm which could have therapeutic benefits. While less is known about the neural circuitry that underlies overexpectation, several studies implicate the central amygdala [47, 48]. While the substrates of extinction and overexpectation might be expected to overlap thus pointing to additional candidate regions, they can also be dissociated at the level of the infralimbic cortex [49]. Which regions are important for the effects of NA in this task should be explored in future studies. Further, as there is some evidence that the LC can corelease NA and dopamine [50, 51], experiments using sensors specific to each (e.g., GRAB_NE_ or GRAB_DA_) [52, 53] potentially alongside experiments that inhibit NA or dopamine receptors could isolate the contribution of each neuromodulator to learning in extinction and overexpectation paradigms.

Finally, studies that directly measure the LC response when a prediction error is introduced would give insight into how the endogenous NA system is engaged by prediction errors. Further, while here, we find evidence that the LC is important for two types of learning driven by negative prediction error, future studies should examine whether the LC has a general role in prediction error and is also involved in learning where that error is positive (i.e. reward is greater than expected) or whether it’s role is specific to negative error, potentially complementing the established role of dopamine in signalling positive prediction error [54–56].

The ability to predict significant events is highly adaptive. It is also essential to be able to update previously learned associations when conditions change. The data here demonstrate that activity of the LC is important for learning to reduce responding in both extinction and overexpectation paradigms. Understanding how the brain updates previous learning to reduce the acquired power of predictive stimuli is important for developing more effective therapies that aim to reduce the influence of such stimuli and to do so with more lasting effects.

## Supplementary information

Dose-Response Testing

## Data Availability

Raw data is publicly available through the University of Toronto’s data repository: ‘locus coeruleus inactivation impairs extinction and overexpectation’, 10.5683/SP3/VDGTGO, Borealis, V2.
