# Supplementary material for: Alpha-2 agonism in the locus coeruleus impairs learning driven by negative prediction error
Source: Neuropsychopharmacology. 2025 Apr 13;50(7):1186–93. doi: 10.1038/s41386-025-02092-5 (PMC12089483; doi:10.1038/s41386-025-02092-5)
Supplement: Supplementary file 1 — Dose-Response Testing [file 41386_2025_2092_MOESM1_ESM.docx]

**Supplemental Materials**

**Dose Response Testing**

To establish an effective dose of clonidine that did not produce sedative effects, we examined the effects of clonidine on reinforced lever-pressing. Rats (n = 8) were trained to lever press for a food pellet reward during stimulus presentations on a continuous reinforcement schedule. The effects of a LC-targeted infusion of 0.2 μL of sterile saline, or 0.6, 1.2, or 2.4 μg of the alpha-2 adrenergic receptor agonist, clonidine (clonidine hydrochloride; MW: 266.55 g/mol; Sigma-Aldrich) dissolved in 0.2 μL of sterile saline on reinforced lever-pressing were assessed within subjects across days according to a Latin square design. A day without infusions was given between doses to avoid any cumulative drug effects. Based on these results, we proceeded with a dose of 0.6 μg of clonidine for the following tests (see Figure S1).


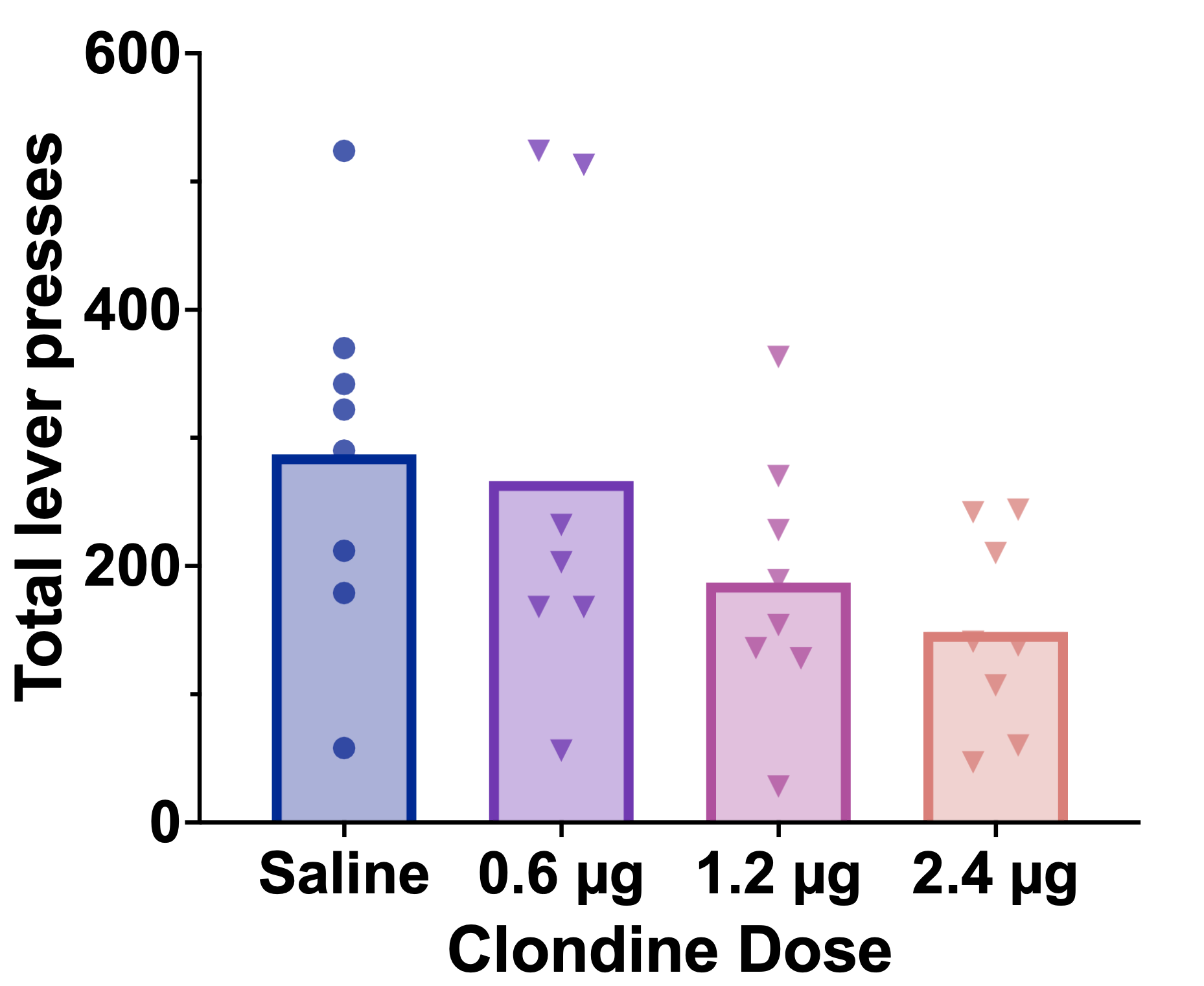


**Supplemental Figure 1. An 0.6 μg dose of clonidine in the LC does not reduce overall responding.** Before conducting our primary experiments, we sought to identify an appropriate dose of clonidine that suppresses noradrenergic function in the LC but was not generally deleterious to food reward-driven behaviours. We examined the effects of an LC-targeted infusion of the alpha-2 adrenergic receptor agonist, clonidine (sterile saline, 0.6, 1.2, or 2.4 μg) on food reward-driven lever pressing. We identified 0.6 μg as the highest dose that did not produce suppression of lever pressing behaviours. Bars represent group means, with individual data points plotted over top
